# IR.WoT: An Architecture and Vision for a Unified Web of Things Search Engine

**DOI:** 10.3390/s24113302

**Published:** 2024-05-22

**Authors:** Cristyan Manta-Caro, Juan M. Fernández-Luna

**Affiliations:** Department of Computer Science and AI, University of Granada, 18014 Granada, Spain; cristyanmanta@correo.ugr.es

**Keywords:** Web of Things (WoT), Internet of Things (IoT), information retrieval, search engines

## Abstract

The revolution of the Internet of Things (IoT) and the Web of Things (WoT) has brought new opportunities and challenges for the information retrieval (IR) field. The exponential number of interconnected physical objects and real-time data acquisition requires new approaches and architectures for IR systems. Research and prototypes can be crucial in designing and developing new systems and refining architectures for IR in the WoT. This paper proposes a unified and holistic approach for IR in the WoT, called IR.WoT. The proposed system contemplates the critical indexing, scoring, and presentation stages applied to some smart cities’ use cases and scenarios. Overall, this paper describes the research, architecture, and vision for advancing the field of IR in the WoT and addresses some of the remaining challenges and opportunities in this exciting area. The article also describes the design considerations, cloud implementation, and experimentation based on a simulated collection of synthetic XML documents with technical efficiency measures. The experimentation results show promising outcomes, whereas further studies are required to improve IR.WoT effectiveness, considering the WoT dynamic characteristics and, more importantly, the heterogeneity and divergence of WoT modeling proposals in the IR domain.

## 1. Introduction

The Internet of Things (IoT) and Web of Things (WoT) are revolutionizing how we interact with the physical world. Miniature-sensor-equipped devices embedded in everyday objects are seamlessly integrated with cloud-based services. This powerful combination, often referred to as the Cloud of Things (CoT) [1,2], overcomes limitations of resource-constrained devices by leveraging cloud processing and storage. These “intelligent things” act as inputs and outputs, feeding into and utilizing globally distributed services. Similarly, Ref. [3] proposes the integration of WoT and edge computing, comparing functionalities, categorizing edge computing based on different architectures, and discussing their challenges and advantages. This convergence presents exciting opportunities for information retrieval (IR); while cloud-based services (XaaS) empower dynamic and scalable WoT applications, they also introduce new challenges for IR systems. The sheer volume and real-time nature of data generated by the WoT demand innovative approaches to IR. Traditional IR systems struggle to keep pace with this dynamic environment.

A New Frontier for IR: The need for synthetic testing and new approaches. The highly dynamic nature of the WoT, with its constantly changing data streams, requires a paradigm shift in IR systems. We need to move beyond existing methods and design new search engines specifically tailored to the WoT. These engines should allow users to discover “things” and “sensors” [4] retrieve information about their features and services, and interact with them effectively. Current IR systems lack the capabilities to handle the complexities of the WoT [5]. To address this gap, we utilize a synthetic collection of real-time IR tests that mimics the behavior of the WoT. This simulated environment will enable us to evaluate existing IR systems and explore new indexing strategies, real-time techniques, and data structures specifically designed for the WoT. This research introduces a novel IR system for the WoT, named IR.WoT. By leveraging some of the capabilities of the CoT paradigm, IR.WoT offers a unified and holistic approach to IR in this dynamic ecosystem. The IR.WoT system tackles the challenges of searching large amounts of data from the IoT/WoT. It aims to deliver efficient search functionalities for various data types and search scopes. The system consists of several interacting modules that collaborate to facilitate searching within the WoT environment; we present design considerations for every stage of the retrieval process. Users can submit queries through an interface to specify keywords, locations, time frames, and data types of interest. The retrieved data are ranked based on their relevance to the user’s query and presented in a user-friendly format. The IR.WoT system is designed to be modular and can be deployed in the cloud.

The following sections will explore the background and related work in IR and WoT (Section 2), delve into the detailed architecture of IR.WoT (Section 3), discuss its design considerations and cloud implementation (Section 4), evaluate its effectiveness using simulated data (Section 5), explore remaining challenges and opportunities (Section 6), and conclude with future research directions (Section 6).

## 2. WoTSE: Background and Related Work

The proliferation of internet-connected devices, forming the vast IoT, has fundamentally changed how we interact with the physical world. Billions of devices generate a constant data stream, promising a future filled with groundbreaking applications and services. However, this potential is hampered by the sheer volume and heterogeneity of these devices. Often lacking standardized communication protocols for higher demands [6,7], which shall allow smooth interoperability between mixed platforms and shall be prepared for future requirements with high data rates, long range, and high bandwidth.

IR is a crucial component of the WoT as it enables devices/users to efficiently locate and acquire the required information. With an ever-growing number of sensors and devices collecting vast amounts of data, IR techniques have become indispensable in filtering and organizing these data. These algorithms help identify the most crucial data based on a device’s specific function, which helps optimize resource consumption and streamline decision making within the IoT/WoT. Future research could focus on developing more flexible and adaptive IR algorithms to better accommodate the constantly changing needs of IoT and WoT.

This section explores the key concepts behind the WoT as a powerful solution that addresses these search challenges. By leveraging familiar web technologies and establishing standardized descriptions of physical objects, WoT acts as a bridge between the physical and digital worlds. Imagine searching for a specific type of sensor to seamlessly integrate into your project, or discovering a smart coffee maker that effortlessly works with your voice assistant—WoT paves the way for these intuitive search functionalities within the ever-expanding IoT landscape.

### 2.1. WoTSE-Related Key Concepts

- Internet of Things (IoT): A network of physical objects, or “things” embedded with electronics, software, sensors, and actuators. These things connect to the internet to collect and exchange data. IoT applications range from simple tasks like monitoring room temperature to complex systems like managing self-driving car networks. Formal and extended definitions can be found in ITU-T (https://www.itu.int/ITU-T/recommendations/rec.aspx?rec=y.2060 accessed on 1 March 2024) and IETF (https://www.ietf.org/topics/iot/ accessed on 1 March 2024).
- Web of Things (WoT): An extension of the web that allows interaction with physical objects through standardized web technologies like HTTP, REST, XML [8], and JSON [9]. WoT provides an abstraction layer, creating a virtual representation of a physical object called a “Thing Description”. This description can be accessed and interacted with using web protocols. This enables applications to consistently discover and interact with a wide range of IoT devices, regardless of the underlying hardware or communication protocols. The term “Web of Things”was used for the first time in 2007 by David Raggett at the UWE Web Developers Conference in Bristol (United Kingdom) according to several specialized media on the subject (https://www.w3.org/People/Raggett/ accessed on 1 March 2024).
- Thing Description: A standardized format for describing the capabilities and properties of a physical object in the WoT. A Thing Description typically includes information such as the object’s type, sensors and actuators, the data it collects, and actions it performs. This standardization allows different applications and devices to consistently understand and interact with the object (https://www.w3.org/TR/architecture-wot/ accessed on 1 March 2024).
- Thing Model: A conceptual representation of a physical object in the WoT. Think of a Thing Model as a blueprint for creating Thing Descriptions. It defines the essential characteristics and functionalities a specific type of object should have in the WoT.
- WoT Architecture: A set of principles and guidelines for designing and implementing WoT systems. The WoT architecture typically includes components like Thing Descriptions, protocols for device-to-application communication, and mechanisms for device discovery and registration.
- WoT Discovery: The process of finding and identifying WoT resources on the Web. WoT discovery protocols allow applications to search for devices based on their capabilities or location.
- WoT Security and Privacy: The set of measures and techniques used to protect WoT systems from unauthorized access, use, or modification. WoT security is crucial as it ensures the privacy and integrity of data collected by IoT devices and services provided through them [10].
- WoT Search Engine (WoTSE): A specialized search engine designed to find and discover WoT resources on the Web [5,11]. Similar to how traditional search engines index websites, WoTSEs index Thing Descriptions or other metadata associated with WoT devices. This allows developers and users to find relevant IoT devices based on their functionalities and capabilities.

### 2.2. Challenges of Traditional Web Search in the WoT Context

Traditional IR approaches used in web search are not directly applicable to the Web of Things (WoT) due to several key limitations:

- Focus on Textual Content: Traditional search engines primarily rely on textual content analysis to index and rank web pages. However, WoT resources often lack rich textual descriptions. Sensors and actuators communicate data streams that require specialized indexing techniques.
- Limited Understanding of Thing Functionalities: Web search engines struggle to understand the functionalities and capabilities of physical objects represented in WoT. Current web search offers limited ways to search based on sensor types, data formats, or actions supported by a device.
- Lack of Standardized Metadata: Traditional search engines rely on metadata embedded in web pages. WoT currently lacks standardized metadata descriptions for physical objects. This makes it difficult to discover devices based on specific functionalities or data characteristics.

The highly dynamic nature of the WoT environment further amplifies these challenges:

- Real-Time Data and State Changes: WoT abstracts a large number of real-world objects that continuously produce data streams. Traditional IR systems are not designed to handle this level of dynamism and require frequent updates to maintain accurate search results.
- Scalability: The anticipated growth of billions of interconnected devices in the WoT necessitates scalable search engine architectures to handle the massive amount of data and information generated.
- Evolving Search Requirements: As the WoT landscape matures, user search needs will evolve beyond basic keyword searches. Users will require advanced functionalities to discover devices based on sensor types, data formats, real-time data characteristics, and desired functionalities within their IoT applications.
- Security, Privacy, and Trust: WoTSEs shall balance powerful search functionalities with user privacy [12]. Data exposure from queries and semantic inferences from sensor data raise concerns. Mitigating these requires minimizing data collection, exploring decentralized architectures, and robust security protocols. Building trust hinges on user control and adherence to evolving WoT security standards [13].

While previous research has explored IR in the context of IoT (e.g., Christophe et al., 2011) [14], it often lacks a focus on the unique challenges of searching within the Web of Things (WoT) environment. Iggena et al. (2021) [15] emphasize the need for more comprehensive WoT search solutions that address device heterogeneity, diverse data types, and user-friendly search functionalities.

Semantic enrichment techniques have emerged as a potential solution to overcome interoperability challenges in IoT/WoT. Cimmino et al. (2020) present eWoT [16], a system that leverages the W3C Thing Description (TD) model but extends it with semantic enrichment through translation to RDF triplestores. However, the authors highlight limitations in the expressiveness of standard TDs in handling the variety of IoT devices. Recently, Mili-Rodin (2022) [17] present WOTMAS2E, a WoTSE based on multiple agents, where the ordering process is based on the probability that a sensor is in certain states, weighted by the number of entities and states, where the process is directly integrating the indexing agent.

Recent studies like that by Llopis et al. (2023) [18] explore additional challenges, including device variety, complex device operations, and energy-wasting search methods. Llopis et al. propose a deep learning-based recommender system utilizing the WoT paradigm and a Transformer model to match user queries in natural language with relevant devices or services within Cyber–Physical Systems. Their work demonstrates promising results for finding relevant devices.

### 2.3. WoT Conceptual Model

As a precursor to WoT models, we must highlight the research of [19]. Guinard defines a mechanism for describing things based on microformats called the “Smart Things Metadata” model, which focuses on the problem of describing the services provided by things and covers essential information for searching for things, functionalities, and services. Furthermore, today, the contribution of the W3C community to the standardization efforts of Thing Description models is well known. The TD W3C thing description model consists of a set of semantic metadata for the thing itself, an interaction model that brings together the properties, actions, and events of WoT to make the models understandable by machines and characteristics for Web linking, providing the ability to express relationships between things. The W3C TD model provides a formal mechanism to describe the interfaces the IoT infrastructure provides and its services, independent of the protocol implementation. However, the thing description models and data formats currently used are diverse and numerous in the different IoT/WoT proposals.

It should be noted that the different modeling proposals in IoT|WoT can vary in “Centricity”, such as in data, sensors, or other things. Just a minority of works are adopting the TD W3C standard model. On the other hand, it is essential to note that the proposals with a focus on data or sensors do not have a layer of abstraction from the real world in the model of the things but instead focus on data collection on variables of interest of phenomena, in the real world and its treatment.

These two paradigms, thing-centric and data/sensor-centric, are complementary and traditionally used for simple categorization. Other classifications and taxonomies have arisen [20,21] considering another umbrella of relationship and centricity, such as things/sensors, services/data, or social/parental relationships. In a previous work [22], we proposed a WoT model that presents a preliminary conceptualization through the abstraction of the real world, together with a WoT representation that was semantically enriched using XML. This model considers spatial and temporal contexts and establishes a model that balances the representation of spatial context and dynamic representations of things and sensors over XSD schemes for later creating dynamic XML representations of IoT/WoT entities.

As a final discussion, it is important to note that in this proposal the description of things is based on three special elements: events, properties, and actions. However, it is also possible to see these from the dimensions of capacities, functionalities, and context, as they are presented in other research. Furthermore, the thing can be seen as an intelligent agent, created with a description and a corresponding behavior. We emphasize the fact that our proposal is based on enriching WoT with XML tags at the level of the WoT semantic model. We also emphasize that the WoT imposes dynamic factors that affect both the effectiveness and efficiency of IR systems.

## 3. Proposed System: IR.WoT Architecture

**Figure 1:**
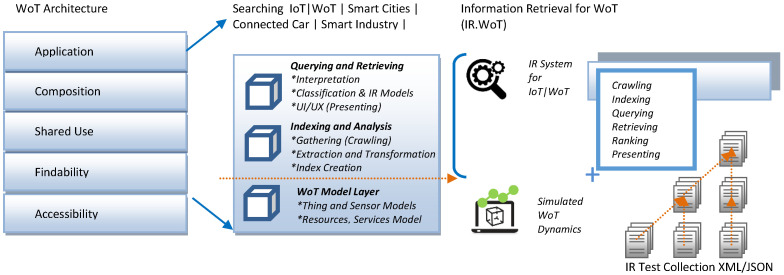
WoT modular architecture and layers.

**Figure 2:**
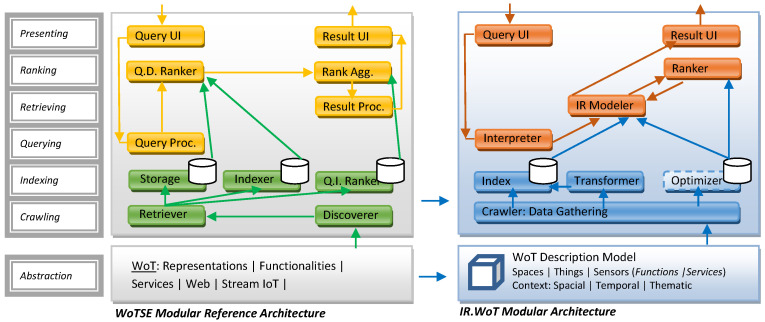
WoT modular architecture styles.

The IR.WoT (Information Retrieval for Web of Things) system tackles IR challenges arising from the vast and heterogeneous data landscape of the Internet of Things (IoT). It aims to deliver efficient and scalable search functionalities for diverse data types, search scopes, and resource-constrained devices. Guinard’s doctoral thesis [19] lays the groundwork for the W3C WoT architecture. It proposes a five-layer architecture with a dedicated searchability layer for discovering relevant information about things, their exposed services, and related applications. Our IR.WoT system aligns with this searchability layer, as depicted in Figure 1 and Figure 2, which also compares with the reference modular architecture in [23].

The IR.WoT architecture, as shown in Figure 1, consists of several interacting modules that collaborate to facilitate IR within the WoT environment. In the illustration, the searching functionalities of a WoTSE could be mapped to the application or findability layers of WoT Architecture:

- Crawling (Data Ingestion/Preprocessing): This module is responsible for collecting data from various WoT devices and sensors, handling different data formats and protocols. In our implementation, an event-driven simulation mechanism is used to inject synthetic WoT-like data. This module also prepares the ingested data for indexing based on the data type. This includes cleaning, normalization, and feature extraction.
- Indexing: This module processes the preprocessed data and builds searchable indexes for efficient retrieval. Indexing techniques may vary depending on the data type (e.g., full-text indexing for textual data, spatial indexing for location-based searches). A more detailed description of the indexing mechanism is provided in the next section.
- Querying: This module allows users to submit queries through an interface. The interface facilitates specifying keywords, locations, time frames, and data types of interest. The design considerations section provides details regarding the graphical user interface (GUI).
- Retrieving and Ranking: This module ranks the retrieved data based on its relevance to the user’s query. The ranking algorithm considers factors such as keyword matching, spatial proximity, and temporal relevance. We employ a base algorithm with extensions inspired by BM25.
- Presentation (Query and Results UI): This module presents the search results in a user-friendly format. This can include raw sensor data, sensor descriptions, or information about physical objects associated with the sensors. This is also responsible for capturing the user information needs in the form of queries.

### 3.1. IR.WoT General System Functionalities

The search engine empowers users to explore the vast WoT landscape through intuitive search queries. Users can target specific sensor data, locate desired sensors, or discover physical objects (things) within the environment. It seamlessly handles various data types, including static sensor readings and dynamic real-time data streams.

The search offers flexibility, allowing users to specify constraints based on content, location (spatial), and time (temporal). Furthermore, it supports context awareness by considering spatiotemporal variables during the search process. Additionally, users can leverage powerful filtering capabilities to refine their search results based on things/sensor actions, properties, and events associated with the data.

### 3.2. Evolutionary Directions and Engineering

The modular architecture proposed by Tran et al. (2018) [23] and the component-based framework for IoT Search Engines (IoTSEs) by the same authors in 2019 [23] offers a solid foundation for designing, building, and evolving Web of Things Search Engines (WoTSEs), while Tran et al. (2018) [24] focus on a modular approach. Tran et al. (2019) [23] delve into the design engineering aspects of such systems. A critical challenge in WoTSE design lies in ranking and retrieving relevant information, especially when dealing with a vast amount of search results. Several works, including those by Tran et al. (2018, 2019) [23,24], propose leveraging natural order by employing ranking mechanisms similar to PageRank or using quality of service (QoS) metrics. Recent advancements like Zhang et al.’s (2021) work [25] explore genetic algorithms for dynamic QoS-based service discovery and selection, demonstrating promising approaches for optimizing search within the dynamic nature of the WoT.

Interestingly, we observe the adaptation of similar internal IR functionalities across different WoT content types. However, design decisions are undeniably influenced by the Thing Description (TD) schema and the underlying semantic mechanisms used for data representation.

From an architectural viewpoint, a significant challenge lies in building a generalized WoTSE capable of performing both local and global searches. Tran et al. (2017) [11] suggest a distributed approach leveraging edge computing and federation to achieve this goal. Liang et al. (2019) [26] echo this notion, advocating for integrating WoTSEs with edge networks and computing techniques to co-design the evolution of Cloud and IoT systems. Nandan et al. (2021) [27] propose an optimized discovery framework that prioritizes faster access and accurate device discovery through data analytics-based object classification.

Standardization efforts are crucial to enable WoTSEs to manage interactions across various Cyber–Physical systems (CPS) and avoid being limited to specific application scenarios. As Skarmeta et al. (2018) [28] highlight, heterogeneity, dynamicity, and scalability remain significant challenges for WoT/IoT systems, demanding ongoing optimization efforts to ensure efficient performance.

Security, privacy, and trust must be fully integrated and addressed within WoTSE design. These have been relatively unexplored issues. Since 2020, we have recognized significant efforts to cover security, privacy, and trust in IoTSE/WoTSE approaches and critical scenarios. For example, Yang et al. [29] propose a Participant Selection Strategy With Privacy Protection (PSSPP) for IoT search. It provides anonymity and blind mechanisms for end-users and requests at query time, along with privacy and identity management. The PSSPP system evaluates participants’ trust value and credibility in the IoT search at mobile crowd-sensing scenarios. In general, security, privacy, and trust concerns are present all over the application-specific scenarios. Only a minority of works are increasing the research attention on adhering to the security, privacy, and trust dimensions of IoTSE/WoTSE. Barclay et al. [30] propose discoverable trusted services in highly dynamic workflows, as those used in 5G/WoT-like scenarios. It provides an enhanced semantic search space for efficient and trusted 5G/WoT-like service discovery. This approach is based on adopting verifiable credentials (VCs) integrated with a vector symbolic architecture (VSA). VCs allow secure information sharing amongst 5G/WoT-like thing peers based on prior service, which is described using SPARQL, RDF and JSON-LD in a cryptographically secure and tamper-proof manner. Current IR systems applied to IoT/WoT often overlook cybersecurity considerations. Manta-Fernandez [12] proposes a novel approach that integrates cybersecurity as a critical dimension within the IR framework for cloud-based IoT search systems. It establishes a theoretical foundation for this approach, followed by a brief survey of relevant research. It proposes a multi-perspective discussion to explore the incorporation of cybersecurity into both IoT and WoT search engine (IoTSE) architectures through the so-called SPTI index parameter.

Introducing Social aspects in IoT/WoT is also a critical factor. Khadir et al. (2020) [31] propose the concept of the Social Web of Things (SoWoT), which emerges from the convergence of Social Networking and WoT/SWoT. Social IoT (SIoT) [32] and WoT represent evolutionary paradigms envisioning social consciousness within smart objects. In SIoT, each “thing” can establish autonomous social relationships with others based on user-defined rules. Nitti et al. (2015) [33] explore a technical definition for SIoT and propose a model for social behavior encompassing various relationship types, such as parental object relationship (POR) and social object relationship (SOR). This model suggests that SIoT systems enable autonomous objects to seek desired services by leveraging their social connections and querying “friends” and “friends of friends” in a distributed manner, mimicking social network principles.

Ma-Liu et al. introduce Progressive Search, a multi-stage search approach for IoT, which offers flexibility in the search process [34]. Pattar et al. leverage this concept in their ProSA system for personalized IoT service discovery [35,36]. They categorize user needs and map them to device properties, enabling coarse-to-fine, near-to-distant, and low-to-high permission searches based on features, location, and security. This aligns with the broader concept of multi-modal search, which incorporates various contextual data types for refined searching beyond just spatial and temporal aspects.

Table 1 summarizes the strengths and limitations of the proposed approach IR.WoT in comparison with other related works. We have considered three of the most significant approaches in the IoTSE/WoTSE field of research. We selected them based on their completeness, significance, and existing detailed information.

## 4. System Implementation: Design Considerations and Cloud Deployment

### 4.1. Design Considerations in the Query and Presentation Module

The Query and Presentation module is responsible for ordering and rendering the weighting and scoring results in an HTML document for the end-user. XML documents, JSON, and structured information may have overlapping elements that must be reorganized to present the information in an easily interpretable format. Since user satisfaction depends on the presentation of results, presentation strategies that eliminate redundancy, maximize similarity in context, and provide the best entry point within the documents must be considered. In the XML Retrieval Evaluation Initiative (INEX) (https://inex.mmci.uni-saarland.de/ accessed on 1 March 2024) evaluation scenarios, these research problems are referred to as focused retrieval, relevance in context, and best in context. Given the universe of research in WoTSE, different strategies have been considered for presenting results in various formats and scopes that complement primary XML retrieval strategies.

#### 4.1.1. Simple and Composite Queries

XML retrieval processing and query languages have evolved from their early origins using XML-QL (https://www.w3.org/TR/1998/NOTE-xml-ql-19980819/ accessed on 1 March 2024), XSLT (https://www.w3.org/TR/xslt-30/ accessed on 1 March 2024), XQL (https://www.w3.org/TandS/QL/QL98/pp/flab.txt accessed on 1 March 2024), to de facto languages still used as XPath (https://www.w3.org/TR/xpath/ accessed on 1 March 2024) and XQuery (https://www.w3.org/TR/xquery-31/ accessed on 1 March 2024). These were later extended to be adapted from data-centric to text-centric with languages such as NEXI [38], ELIXIR [39], XIRQL [40] and XQuery Full-text (https://www.w3.org/TR/xquery-full-text/ accessed on 1 March 2024). Conventionally, two categories of query can be identified as Content-Only (CO) and Content-and-Structure (CAS). The Narrowed Extended XPath I (NEXI) query language developed by the INEX [38] community is based on XPath expressions to access and navigate within the components and elements of the XML document collection. Because the exact containment of elements can be less critical in IR applications, NEXI only supports descendant or auto (//) notation for routes. To specify the classified retrieval, In the IR.WoT user-facing fields and constraints are captured through the query interface and then translated from natural language to NEXI queries. Conventionally, translation can be performed with several methods given the structure and content of the XML documents in the collection. For the proposed model, the following types of EXI queries are used:

- Simple Queries in the form //A[B]
- Composite Queries of the form //A[B]//C[D]

where A is the target XML element of the query to be retrieved, and B is a filter or content constraint (CO). In such a way that a pure query (CO) that retrieved any type of document and XML element within the collection with the filter provided by the user in the main query field would be translated to:

(1) //∗[about(.,co_query)]

As an example, we could cite the general need for information of a person doing tourism in a city that they do not yet know:

(2) //∗[about(.,tourisminBarcelona)andabout(.,bikeriding)]

Restrictions have been considered to the document type whose identification is encapsulated in the “*type*” attribute of the “*vX*” root element of XML documents. So, an attribute filter is set with its corresponding NEXI query:

(3) //vX[@type=virtualSensorandabout(.,co_query)]

(4) //vX[@type=virtualThingandabout(.,co_query)]

(5) //vX[@type=intelligentZoneor@type=smartSpaceandabout(.,co_query)]

For the first case, virtual sensor-type documents/elements related to the content-only query would be retrieved, in the second case only virtual thing-type documents, and finally the NEXI query would consider any type of documents descriptor of zones, spaces and intelligent subspaces. Using the previous example, if the query user wanted only tourist or bicycle rental places related to their query, they would use the structure constraint encapsulated by the document type attribute that belongs to root elements.

We additionally propose both spatial and temporal context constraints. Thus, space and time filters are set through the selection object in the query interface. Conventionally, today’s search engines do not set restriction, which could be translated into framed results anywhere “*anywhere*” and at any time “*anytime*”. However, the WoT paradigm has oriented WoTSE approaches towards a focus on the here “*here*” and now “*now*” [41]. Considering these new elements, the possibility of including arithmetic filters on space and time is created. In our proposal we consider that the current real-time location of the user could be captured by the IR.WoT if it is shared given the GPS global positioning system sensor information on a device or is an environment variable.

Standard queries for spatial and temporal constraints:

(6) //∗[about(.,co_query)and.//event/eventTime<={time}]

(7) //∗[about(.,co_query)and.//property/geocoordinate<={distance}]

In the query interface there are fields for capturing information associated with the construction of structure filters on XML elements, properties, actions, and events. These allow you to explore the structure of XML documents and structure more complex NEXI queries, providing the user with the possibility of richer options in the process of searching for information about a WoT system. In this way, the possibility of finding things, sensors or spaces with particular characteristics is presented, or the possibility of offering services or functionalities in the form of actions or about states in which they are given their history of events.

Queries type of structure and content:

(8) //vX[@type=virtualSensorandabout(.,co_query)]//events[about(.,cas_query)]

(9) //vX[@type=virtualThingandabout(.,co_query)]//actions[about(.,cas_query)]

(10) //vX[@type=smartSpaceandabout(.,co_query)]//properties[about(.,cas_query)]

#### 4.1.2. User Interface—Query UI

**Figure 3:**
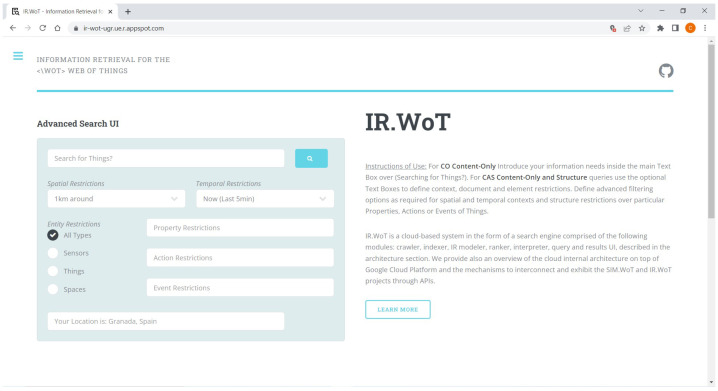
Proposed query interface for IR.WoT.

Figure 3 showcases the proposed user interface (UI) for querying the IR.WoT system. This UI facilitates the creation of both CO and CAS type of queries, allowing users to incorporate spatial and temporal constraints within their searches.

Key Features:

- Main Menu: The UI presents a main menu with hyperlinks to various sections, including:
  – A search bar for entering natural language queries.
  – Structure constraints to filter results based on sensors, things (physical objects), or spaces. This allows users to refine their search based on the type of data they require.
  – The ability to search for XML elements related to contact information for individuals who own or manage elements within the WoT system (e.g., sensors, things, spaces).
- CAS Constraints: The system offers advanced options for specifying CAS constraints on XML elements. These constraints can be applied to properties, actions, and events associated with the data.
- Location Awareness: The IR.WoT system offers location-aware search functionality. By default, it will prompt users to share their current location to center search results geographically. Alternatively, users can choose to assume a specific location (e.g., Granada, Spain) as the search origin.
- Navigation Bar: The left navigation bar provides informative hyperlinks:
  – Information about the underlying scientific research project.
  – Details on the IR system itself.
  – Submenus offering:
    * A “Home” or search interface button.
    * Instructions for using the search engine.
    * An overview of the general IR.WoT system architecture.
    * Advanced configuration options for the search engine (accessible to users with technical expertise). This menu can be collapsed to maximize screen space for the search interface.
- Open-Source Development: The IR.WoT system, along with the associated SIM.WoT discrete event simulator (used to generate synthetic data) is licensed under the General Public License v3.0. The source code is available on GitHub, accessible through the icon in the top right corner of the UI.

#### 4.1.3. User Interface—Results UI

In traditional web search engines, results are displayed in descending order of relevance to the query. However, this method may not be the most efficient for displaying results in the context of WoT, where XML/JSON formats with interdependent nested elements are used. To improve the display of results, three main strategies can be used: focused recovery, relevance in context, and best in context. These strategies aim to minimize information redundancy and provide users with relevant information within the structure of the element’s membership in its corresponding document.

To achieve optimal efficiency and increase user satisfaction rates, it is crucial to consider spatial and temporal contexts in the presentation of search results. Additionally, algorithmic perspective should be taken into account as these tasks could influence each other. Therefore, our proposal assigns a priority value to each presentation strategy to ensure consistency across all query types. By implementing these strategies, we can create a more effective and user-friendly interface for WoT search engines.

**Figure 4:**
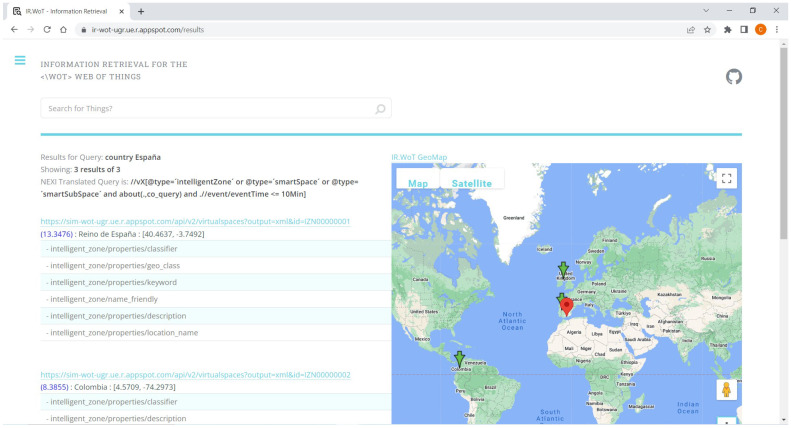
Proposed UI interface for IR.WoT results.

Figure 4 illustrates an example of the IR.WoT system results interface. Three (3) important areas can be seen in it:

- General Information of Results. The user is provided with the following information:
  – The query terms entered by the user are paraphrased.
  – Number of Results presented on the page and the total number of matches. Or if not, a message if no match is found.
  – An indication of the translation to NEXI of the constraints entered by the user.
- Ordered List of Results. Each outcome item is presented with the following detailed information.
  – Hyperlink to the matching XML document.
  – The relevance value for each document, this for practical and prototyping purposes only.
  – Short descriptive name of the corresponding entity.
  – Geographic coordinates of the entity corresponding to query time.
  – General status of the entity (virtual things and sensors) at query time.
  – Sorted list of internal XML elements matching the query from most to least relevant.
- Results Location Map.
  – Center of the map corresponds to the current location of the user or query.
  – Geo-localized markers for each of the results.
  – Search radius selected by the user in the query.

### 4.2. Design Considerations in the Indexing and Analysis Module

IR solutions based on static indexes are not well-suited for the dynamism of the Web of Things (WoT). Recent research in XML retrieval has shown that structured information in the form of XML/JSON documents with hierarchies and implicit relationships has impacted the way XML mechanisms are being developed. However, an adaptation of fundamental components is required to address the characteristics of the WoT. A model based on XML is proposed, where the indexer will be based on data structures capable of efficiently providing insertion, modification, deletion, and other operations. A study evaluating different data structure options for constructing an index schema for XML is presented in [42]. Inverted indices are the basis of most IR systems. Inverted index schema consists of a term dictionary linked to publication lists that ultimately contain information on the positioning of the term in the collection and in the document along with occurrence statistics.

Maintenance of inverted index scheme is essential to keep the index up-to-date. Different maintenance strategies exist, including semi-static, incremental, and dynamic ones. However, dynamic maintenance strategy has not been well-studied or implemented in the classic XML indexing mechanisms [43,44]. In conclusion, an indexing system for WoT needs to consider additional indexes for the efficient storage of information of IoT resources, things, and the WoT system in general. Further research is needed to develop strategies for dynamic maintenance of inverted index schema in the context of the WoT.

**Figure 5:**
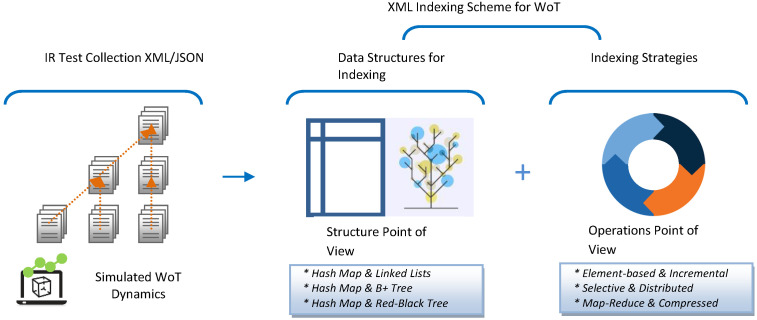
Indexing scheme for IR.WoT.

As shown in Figure 5, we propose to analyze the combination of three data structures by applying three combined XML dynamic strategies. In the following subsections, we will describe the decision behind the combinations in structure and operations from theoretical and pragmatic perspectives. A study is presented in [42] in terms of evaluating the performance of different data structure options for constructing an indexing scheme for XML. It is a starting point for discussing data structures as bricks for a dynamic WoT indexing scheme.

As mentioned above, the dictionary that makes up the inverted index can be stored in a *hash* table or similar structure, and the list of posts for each term *t* can be stored in a fixed-length array structure $l_{t}$.

**Figure 6:**
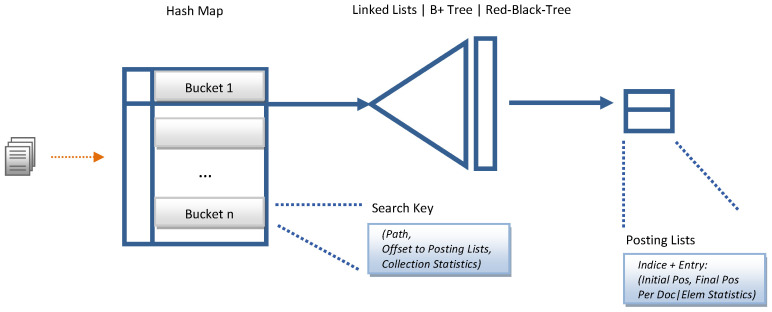
Index structure proposal for IR.WoT.

**Figure 7:**
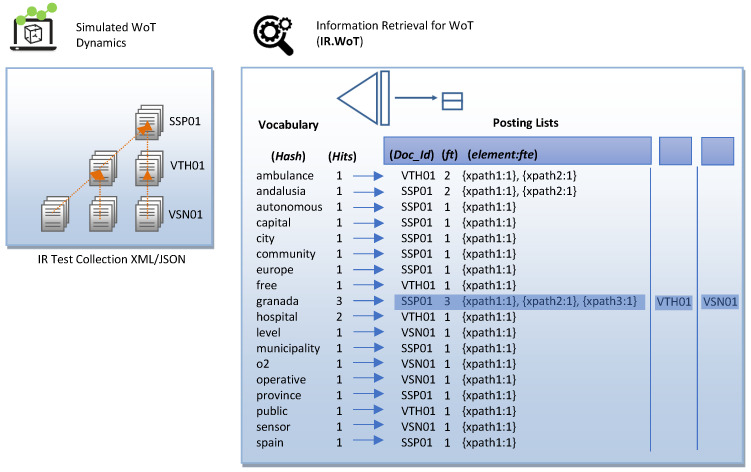
Index construction example: structure.

**Figure 8:**
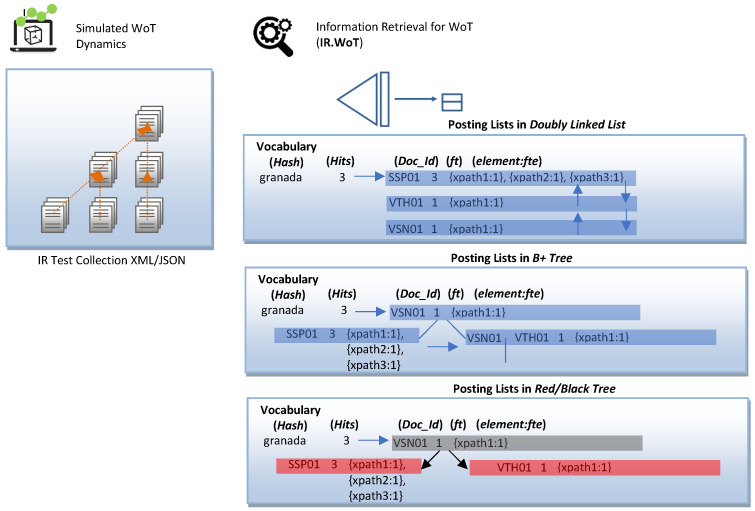
Example of index construction: publication lists.

From an architectural point of view, XML indexing structures are classified based on the type of information stored in the dictionary, taking on the role of a search key. The goal behind data structures is to reduce the search space to increase the performance of the IR mechanism. We have selected the structural join architecture because our IR.WoT system handles direct CO content queries and conditional queries on CAS content and structure provided by the WoT user in natural language. The search critical stores text and numerical data, as shown in Figure 6. The time complexity analysis of the combined data structures is shown in Table 2. We provide sample figures on the index construction structure in Figure 7 and for the posting list in Figure 8.

We have decided to base the implementation of our indexing scheme on Python 3.7.4, as it provides powerful processing tools while keeping the code simple and readable. First of all, we use the module *xml.etree.ElementTree* module (https://docs.python.org/3.7/library/xml.etree.elementtree.html accessed on 1 March 2024) which implements a simple API and efficient for parsing and creating XML data with built-in *XPath* support. We then initialize combined structures using open source implementations of linked lists, B+ trees, and black-red trees available under MIT licenses at the Python Software Foundation (https://pypi.org/ accessed on 1 March 2024). Finally, for each document in the collection, the indexer constructs and updates the schema-dependent inverted index. We use the reference inverted index algorithm in [45] with some adaptations; see Algorithm 1.
**Algorithm 1** Index construction in IR.WoT**Require:** D←XMLDocument  1:**Begin**  2:Initilize complex structures I(s)=0, for all s∈S  3:**repeat**  4:     **for each** XMLelementxe∈D **do**  5:          **if** xeexistsinI **then**  6:                $Posting\;lists\leftarrow xe_{id}$  7:          **else**  8:                I[search_key]←xe  9:          **end if**10:     **end for**11:**until** Collectionisdone(ForeachDinCollecion)**Ensure:** StatsUpdation

The input XML document *D* is represented as an ordered tree with root. Each node in the XML tree mainly contains elements, but may also contain attributes on the elements, and the edges between nodes represent structural relationships such as parent-child or ancestor-descendant.

### 4.3. Design Considerations in the Ranking and Retrieval Module

The XML retrieval model employs an extension of the well-known Okapi BM25 algorithm. In the course of the INEX and in other research forums, a set of applications of the base BM25 algorithm with its modifications for focused, element or textitsnippets [46]. Although alternative models have recently been proposed, such as the approach of Belahyane et al. [47] using graph theory or in Bessai et al. [48] using genetic algorithms, the performance of BM25 on INEX *tracks* has not been significantly surpassed. Furthermore, there is a general consensus to use variations of BM25 to weight characteristics of the XML structure within element and document statistics.

Typically, search tasks focus on returning a relevance-ordered list of XML document elements in response to a user query. It is additionally required that the XML elements do not overlap, that is, that there is no hierarchical relationship between the elements, one contained in the other [49]. XML element retrieval uses the following version of:

(11) $$\mathrm{score}\left(E,Q\right)=\sum_{i=1}^{n}\mathrm{IDF}\left(q_{i}\right)\cdot \frac{f\left(q_{i},E\right)\cdot \left(k_{1}+1\right)}{f\left(q_{i},E\right)+k_{1}\cdot \left(1-b+b\cdot \frac{|e_{E}|}{\mathrm{avgdl}}\right)}$$

where *Q* is a set of query terms, IDF is the ith term weight, $f(q_{i},D)$ is the frequency of terms in an XML element, $e_{E}$ is the length of an XML element of *E*, and avgdl is the average length of a document in the collection. Applying BM25 directly still uses document-level statistics for the IDF calculation. BM25F is an extension that exploits structural information from the XML document. The relevance score for an element is calculated as:

(12) $$\mathrm{score}\left(E,Q\right)=\sum_{t\in q\cap e}\mathrm{IDF}\left(q_{i}\right)\cdot \frac{{\bar{x}}_{e,t}}{K+{\bar{x}}_{e,t}}$$

where ${\bar{x}}_{e,t}$ is the normalized and weighted term frequency and *K* is an adjustable parameter. To obtain ${\bar{x}}_{e,t}$ length normalization is first performed separately for each field *f* associated with an element *e*, producing frequencies of specific normalized terms.

### 4.4. Cloud Deployment Considerations

The source code of IR.WoT is available in the public GitHub repository published as open source under the GPL 3.0 license at (https://github.com/cristyanmanta/ir-wot-ugr accessed on 1 March 2024). This contains the Python project prepared in the development environment *PyCharm Professional Edition* (https://www.jetbrains.com/pycharm/ accessed on 1 March 2024) used under a student license package. The IR.WoT proposal contemplates the critical stages of indexing, query interpretation, retrieval, scoring and presentation. This repository contains some design, cloud deployment, and experimentation considerations based on a collection of synthetic XML documents from simulation run in SIM.WoT.

For installation on Google Cloud App Engine, the standard environment guidelines for Python are used, the code can be automatically deployed using the Management SDK with the command *gcloud app deploy*. For local use, the main Python file is executed through the *python main.py* console.

The documentation pertaining to the IR.WoT system RESTful API contains a collection of calls created to interact with the IR.WoT search engine. This provides API calls to:

- Search in the Web of Things (WoT).
- Run CO and CAS queries in WoT.
- Run experimental framework with IR.WoT.
- Collect IR.WoT experimental statistics.

Whose definition can be consulted in document *Swagger*, see (https://app.swaggerhub.com/apis/cristyanmanta/ir_wot/v1_0/ accessed on 1 March 2024). Technically talking, a first classic crawling approach is used, where a stack of XML documents to be indexed is built from a set of seed URIs/URLs. From this stack, the indexer begins index creation starting at simulation time zero. Additionally, for each simulation event, a modification of the XML documents is notified, which concludes in a scheduled re-indexing of the modified XML document.

## 5. Experimentation and Evaluation

This chapter describes the set of experiments designed for the evaluation of IR systems for WoT. The experiments are carried out using the cloud services of *Google Cloud Platform* to isolate the IR.WoT search engine from the SIM.WoT simulation engine (the simulation engine is described in [50]) on different projects. The goal of this isolation is to avoid errors in measuring performance metrics. We have decided to connect our proposed IR.WoT architecture through a RESTful API, thus emulating a realistic WoT scenario where the search engine crawls things over HTTP(S). We have used an IR experimental XML-based collection of dynamic documents simulated with real-time data, some statistics of the collection are listed in Table 3.

Evaluating a WoTSE is a complex process that requires consideration of several factors. Furthermore, as stated previously, no standard agreement exists on evaluating them. First, the evaluation process should consider the relevance and accuracy of the search results generated by the engine. However, it is well-known that there is a lack of datasets judged by experts’ content-labeled data on relevancy. The process involves analyzing the engine’s ability to retrieve and rank relevant WoT resources based on the user’s search query. Second, the evaluation should consider the usability and user experience of the search engine, including its interface design and ease of use. Third, the evaluation should consider the engine’s performance and scalability, such as its ability to handle a large volume of search queries and provide results in a timely and efficient manner. Overall, a comprehensive evaluation of a WoT search engine should consider all these factors to ensure that the engine meets the user’s needs and expectations.

### 5.1. Evaluation in Time and Space Performance

**Figure 9:**
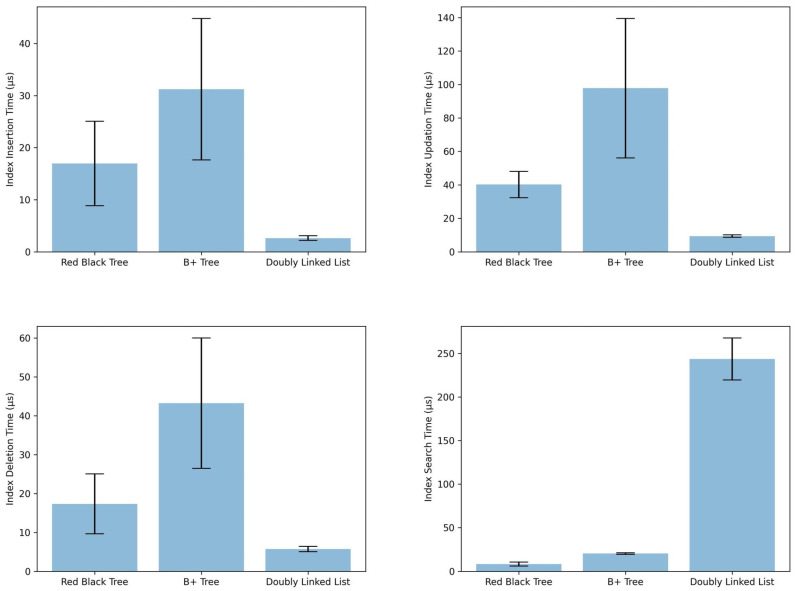
Time results in index operations.

This evaluation set focuses on the performance of the proposed data structures for indexing WoT data. We aim to experimentally validate their time complexity for various operations; see Figure 9:

- Insertion: We measure the average time required to insert new data into the index structures.
- Updation: We evaluate the average time required to modify existing data within the indexes.
- Deletion: We measure the average time to delete data from the indexes.
- Search: We evaluate the average time required to perform search queries using the different index structures.

**Figure 10:**
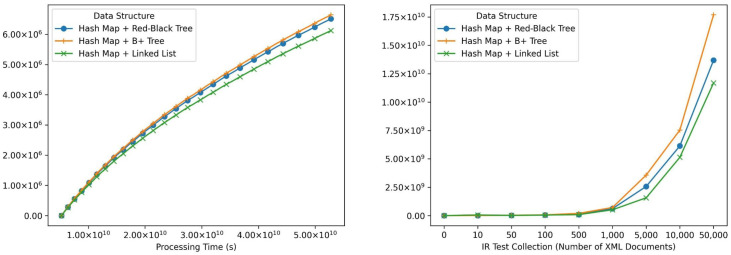
Size results in index creation.

Additionally, we analyze the space complexity of the proposed data structures, see Figure 10; while all theoretically exhibit a worst-case scenario of O(n), we conduct an experiment to measure the memory footprint of each index type for a more practical understanding.

### 5.2. Usability and User Experience Evaluation

**Figure 11:**
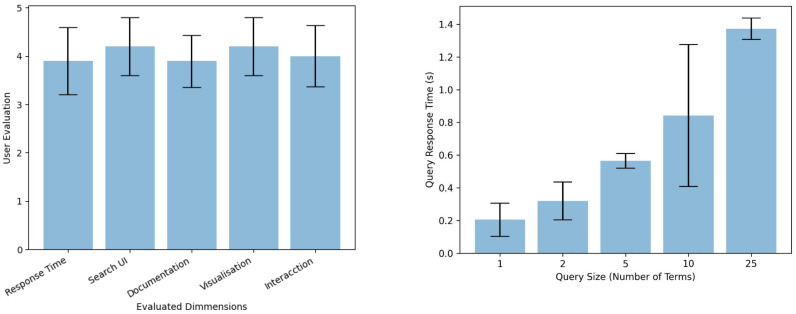
User evaluation on IR.WoT search engine.

This set of experiments evaluates the overall performance of the IR.WoT system. We aim to measure the average response time for processing a series of synthetic queries with varying keyword lengths. We leverage a user-oriented web search engine evaluation model adapted from Su et al. (2003) [51]. This model incorporates user relevance judgments, where users assess the retrieved results based on their own information needs. See Figure 11.

#### 5.2.1. Test and Control Procedures

For the purposes of applying the IR.WoT search engine evaluation model by a group of end-users, direct access is provided to users through the public instance (at: https://ir-wot-ugr.ue.r.appspot.com/ accessed on 1 March 2024). The test suite is conducted using forms and a procedural guide described below in this subsection. Both the sequence and timing of tasks and measurements are important to ensure consistency of assessment. Three test sites have been selected (Universidad Distrital Francisco José de Caldas, Politécnico Grancolombiano) with the participation of five (5) participants for each site.

#### 5.2.2. User Testing Session I

1. Complete a participation consent form and a background and user experience questionnaire. It is used as the base model of the information and informed consent sheet for the study participants.
2. Provide information about the user’s information needs and search requirements in a search form.
3. Receive tutorial session on the use of IR.WoT.
4. Perform searches on the user’s own information problem in the order provided.
5. Use the online instructions by clicking on the corresponding menus.
6. Obtain a digital copy of the search results of a predetermined size;
7. Complete an online questionnaire regarding user satisfaction regarding IR.WoT search engine features and interaction.
8. Repeat steps (4) to (7) until all searches are tested.

#### 5.2.3. User Testing Session II

1. Evaluate the relevance of search results according to a set of delivered judgment guidelines.
2. Select and sort items from search results according to the instructions provided.
3. Participate in a post-search interview to provide feedback on the search process, IR system, and overall engine performance.

### 5.3. Experimentation Report and Analysis of Results

The creation time of an index is directly proportional to the size of the test collection (number of documents) and the average size of the XML documents to be indexed. Additionally, the MIME type of the data can also significantly impact indexing time. The MIME type indicates the data format (e.g., text, image, video). Complex formats like images or videos require more processing steps during indexing than plain text, potentially increasing indexing time.

While basic data structures have a constant algorithmic complexity for insertion operations, their implementation in languages like Python, Java, and C can impact practical performance. Since the evaluated implementations correspond to implementations following the object-oriented programming (OOP) paradigm of the B+, Red-Black tree with public libraries of *Pypi*, their performance in time and memory is not optimal. In practice, implementing a *Hash* map and a Double-Linked list has the best time performance. Implementing data structures based on well-known language structures optimizes their execution in the runtime environment.

Data preprocessing steps can also significantly influence indexing performance. Techniques such as stemming and stop-word removal can significantly reduce the volume of data to be indexed. This, in turn, can lead to faster indexing times and potentially smaller index sizes. Additionally, data cleaning steps like handling missing values, correcting inconsistencies, and recognizing different representations of the same entity can improve the accuracy and efficiency of the indexing process.

It is important to analyze the trade-off between preprocessing effort and potential benefits. For scenarios with large, constantly changing data sets, focusing on efficient indexing structures like the proposed Hash + Doubly Linked List might be more beneficial than extensive preprocessing. Conversely, for static data sets where retrieval accuracy is paramount, a more thorough preprocessing approach might be justified.

Based on experiments (see Figure 9), implementing a *Hash* map and a Double-Linked list performs best in time, making it the most suitable structure for WoT scenarios with high-speed collection changes, i.e., for indexing and re-indexing (updating). The implementation of this structure allows insertion operations below 5 µs (2.6 µs average) and update operations below 10 µs (9.3 µs average) in the experiments carried out, that is, approximately 6 × faster than the structure based on *Hash* + Red-Black tree and 12 × faster than the structure based on *Hash* + B+ tree in insertion. Nevertheless, it is important to consider the processing times required by intermediate steps in tree structures to balance or mark branches/leaves, which ultimately speed up search operations in practice. It is important to notice search time complexity implications; while *Hash* structures offer constant average-case lookup time O(1), this can degrade to linear time O(n) in the worst case, especially with large datasets. In contrast, Red-Black trees maintain a guaranteed logarithmic search complexity O(logn), ensuring efficient retrieval even for massive collections.

For WoT scenarios with a moderate collection change rate and a high number of entities, the structure composed of *Hash* and a Red-Black tree is the best indexing option for retrieving information in this context. Although the results’ freshness may be impacted due to the cost of insertion, deletion, and update time, exhibiting a shorter search time between the compared structures is the best option for large to massive collections as it requires search operations on a high number of documents in the collection. Since the number of publication lists is directly proportional to the number of entities and, therefore, to the number of descriptor XML documents, the retrieval time will be increased by search operations in the index. In the structure *Hash* + red-black tree O(Log(n)) complexities are achieved in the experiments below 10 µs (8.17 µs average), which is 2.5 × faster than the *Hash* + B+ tree and 30 × faster than *Hash* + *Doubly Linked List*. Therefore, the choice between Hash + Doubly Linked List and Hash + Red-Black Tree hinges on the expected balance between update frequency and search performance. For WoT scenarios with highly dynamic data and frequent updates, the faster insertion/update times of the Hash + Doubly Linked List might outweigh the potential drawback of slightly slower searches. However, for scenarios prioritizing accurate and fast retrieval on large, relatively static data sets, the Hash + Red-Black Tree offers a better overall balance.

The size of the index depends on the size of the dictionary in terms of a number of terms and on the size of the publication lists stored by their corresponding structures in memory. The size of publication lists is a factor in the structure of the XML documents and, therefore, in the depth of the hierarchy or parent–child relationships of the XML elements. To ensure optimal performance, it is vital to store publication lists for read/search operations in a way that is more computationally efficient for retrieval models that will access the index for relevance calculation given a query. In some retrieval models, it is desirable to store in document identification order, while in others, it is important to store collection, document, item, and term statistics (tf*idf) for runtime availability of the relevance score or similarity calculation algorithm.

Concerning to storage resources, traditional constructs of data structures make decisions based on RAM, hard drive, and/or cloud storage. However, memory limitations can force the use of hard disks, impacting system performance; while experiments have been carried out by building the complete index in memory, future work will address the use of cloud storage (in its different forms: NoSQL, Non-relational, objects, documents, blocks) for the construction of distributed indexes. Shared cache technologies in the cloud can also be used.

In conclusion, this report analyzes the impact of data structures on indexing performance in WoT scenarios. For scenarios with high data changes (frequent insertions and updates) and a preference for fast update speeds, a *Hash* + Doubly Linked List structure offers the best performance. However, for scenarios with a moderate update rate but a high number of entities, where efficient retrieval is crucial, a *Hash* + Red-Black Tree provides a superior balance between update times and search complexity. To further optimize retrieval performance, careful consideration should be given to storing publication lists in a format aligned with the specific retrieval models used (e.g., BM25, TF-IDF). Finally, leveraging cloud storage and shared cache technologies can be advantageous for constructing distributed indexes that handle massive data volumes and enable efficient retrieval across geographically dispersed WoT deployments.

## 6. Conclusions and Future Work

The Web of Things (WoT) is a dynamic ecosystem that provides real-time data about the physical world. These data are obtained via sensors that gather information such as geographical location, physical, chemical, or any other type of variables of interest. The dynamic nature of this data, which is updated and deleted constantly, presents challenges for the design and development of new IR systems.

The architecture of an IR system for the IoT, WoT, must be designed with a multi-dimensional focus, balancing effectiveness, efficiency, and adaptability, while high-quality results (effectiveness) and fast response times (efficiency) remain crucial, the dynamic nature of WoT environments necessitates an adaptable architecture. This adaptability should address real-time data streams, heterogeneous data, and context awareness. Recent research advancements in areas like edge computing, machine learning ML, and semantic enhancement can be explored to build an IR system that is not only effective and efficient but also adaptable to the ever-changing dynamics of the WoT landscape.

The IR.WoT system addresses IR challenges in the WoT landscape by providing a modular and scalable architecture. The system considers the unique characteristics of WoT data, such as its heterogeneity, volatility, and resource constraints. Additionally, the design of the query and presentation modules ensures user-friendliness and efficient interaction. By integrating with existing WoT infrastructures, IR.WoT can empower users to discover valuable information within the ever-growing world of the Internet of Things.

The proposed IR system IR.WoT has adapted traditional indexing through hybrid models for retrieving in the dynamic IoT|WoT environment. This demonstrates two essential points: on the one hand, the possibility of adapting existing mechanisms while building on the existing knowledge base; meanwhile, on the other hand, exploring the addition and evolution of new approaches to indexing that address the characteristics of IoT|WoT building an evaluation framework for future development and implementation.

In this work, an IRaaS-type architecture is proposed FOR IR.WOT is a way to take advantage of cloud resources for the implementation of IR mechanisms adapted to WoT and, at the same time, raise the discussion on research into the distribution or not of these IR functions, where computing capacity, storage, network and security are considered fundamental factors. The WoTSE/IoTSE architecture must be around new paradigms that drive new alternatives to building search engines on micro-devices attached to things, or on the edge of the cloud using fog computing, *Fog Computing* distributing the stages closer to the end-user changing the traditional geo-distribution of stages in large Datacentres.

Conventional IR mechanisms are not always suitable for WoT, as they were developed for a static web. The WoT paradigm allows not only for obtaining information about the state, properties, and events of things but also for interacting through actions on things and their environment.

The WoT model consists of sensors at the lowest layer, with a series of overlapping or interposed layers depending on the investigation’s purpose. The central layer is a real-world feature abstraction layer that exposes a considerable number of alternatives for description and representation. These models and/or languages for describing things range from metadata, microformats, microdata to ontologies. JSON/XML standards are being selected for the construction of representation schema for WoT in conjunction with extensions and other varieties of data formats such as JSON-LD.

Standardization bodies and interoperability are necessary standards within scientific research and are required to join forces in the evolution of new paradigms and the ecosystem of applications and services around them. The proposed WoT model considers and is characterized by a vision of the real world that pays importance to the spatiotemporal context, adding relationships between things, sensors, and spaces.

There are numerous and promising efforts by organizations such as W3C, IEEE, ISO, IETF, ITU-T, and OpenGIS, among others, to standardize technologies that aim at the interconnection of the real world and interaction with it through avatars or digital twins. The proposed WoT model is expected to constantly evolve and remain for a longer life cycle. The control of this life cycle can be addressed through standardization; in this way, technologies such as HTTP, CoAP, XML, and EXI, which are well-known web technologies, are and will be continuously evolving by W3C to face these mechanisms.

In conclusion, WoT presents a different dynamic that should be considered in designing and developing new IR systems. The WoT model and description languages become a cornerstone and driver in developing and researching new surrounding systems, including IR. The limitations and challenges faced by the evolution of IR systems must be recognized, mainly due to the scarcity of IoT systems and the same scarcity of data or IoT collections. It is expected that Web and WoT will constantly evolve and that things will remain for a longer life cycle.

### Future Work and Research Directions

In the field of IoT and WoT, several challenges need to be addressed to improve the effectiveness and efficiency of IR systems. One major challenge is the concept heterogeneity, making it difficult to reproduce and evaluate experiments and access and reuse datasets. Exploration of advanced search techniques, such as semantic search and machine learning-based ranking algorithms, to further enhance retrieval accuracy and user satisfaction can be some research directions. All consider common terminology and concepts, homogenizing the path for a common evaluation framework.

Future work should address the limitations identified in the current study regarding data volume and type to enhance the generalizability and real-world applicability of the findings. This can be achieved by leveraging big data techniques and incorporating a wider range of data types that reflect the complexities of real-world Internet of Things (WoT) scenarios. Simulations involving a massive number of entities can be employed to evaluate the scalability and performance of the IR.WoT system under various conditions. This would provide more comprehensive insights into the system’s effectiveness in handling the data volume and variety typically encountered in real-world WoT deployments.

To address the issue of reproducibility, it is essential to build open and public datasets for IoT and WoT and to provide complete experimental details for evaluation purposes. The lack of dataset transparency, evaluation methodology, and system parameters can hinder the reproducibility of experiments. Standardization of evaluation criteria and performance metrics can help compare different IR approaches.

Access to datasets is also challenging, as many domains and data are not open to researchers; while there are some publicly accessible datasets, creating new collections and access to more domains is still needed. Another challenge is the difficulty of reusing modular RI components due to the use of non-typical architectures and interfaces between components. To overcome this challenge, agreed-upon architectures, descriptors, and libraries can help with component reuse.

Security, privacy, and trust are critical challenges for IR in IoT and WoT. Protecting data within these systems should be a top priority for all IR tasks. New lines of research are needed to address these challenges and improve the overall security, privacy, and trust of IR systems. For IoTSE/WoTSE in general, present and future considerations include:

- Data Minimization: Minimizing the amount of data collected and stored is crucial. WoTSEs should focus on retrieving only the data necessary to fulfill user queries.
- Decentralized Architectures: Centralized user data storage creates a single point of failure and a privacy risk. Exploring decentralized architectures can distribute trust and potentially enhance privacy.
- Dynamic Consent Management: Users should have granular control over what data is collected, used, and for how long. Fine-grained consent mechanisms are essential.

Security is paramount for building trust in WoTSEs. Strong authentication and authorization mechanisms are needed to prevent unauthorized access and manipulation of search queries and data. Standardization efforts on data formats, privacy controls, and security protocols are crucial for ensuring interoperability and trust in a rapidly evolving WoT landscape.

In future work, it is important to address the representation of the spatial context in simulation and in the WoT architecture itself. spatiotemporal context can be enhanced by utilizing multi-layered geographic information systems (GIS) and a spatial subdivision approach. Additionally, using semantic models based on RDF, RDFa, and OWL ontologies may allow a homogeneous approach across application domains to exchange information in the WoT ecosystem. These approaches can help provide relevant, highly dynamic, and highly interactive information to users.

## Figures and Tables

**Table 1:** Strengths and limitations of IR.WoT system compared to other IoTSE/WoTSE works.

Feature	IR.WoT	WoTmAS2E [17]; Mili-Rodin (2022)	eWoT [16]; Cimmino et al. (2020)	[23]; Tran et al. (2018, 2019)	WOTS2E [37]; Kamilaris et al. (2016)
**Strengths**					
Data Handling	Handles some diverse data types	Not explicitly addressed	Leverages semantics for heterogeneous data interoperability	May not support all data types	Supports diverse data types through progressive search approach
Search Flexibility	Allows for spatial, temporal, and thematic, content-based search	Spatial, temporal, and content-based search	Enables semantic search based on ontologies	Keyword-based search with ranking based on natural order	Semantic search with focus on geospatial aspects
Context Awareness	Considers spatiotemporal and thematic aspects during search	Considers spatiotemporal aspects during search	Can potentially leverage semantics for richer context awareness	Lacks consideration of specific contextual factors	May consider contextual factors through progressive search approach
Ranking and Retrieval	Employs BM25-inspired ranking for relevant IR	Not explicitly addressed	Not explicitly addressed	Utilizes ranking based on natural order or QoS metrics	Semantic similarity measures for ranking sensor data and actuator capabilities
Modular Architecture	Facilitates easier integration and future enhancements	Modular architecture with focus on agent communication	Layered-based approach which relies on SPARQL	Component-based framework	Component-based framework
Security and Privacy	Acknowledges the importance of security and privacy and trust, not addressed	Security and privacy not explicitly addressed	Security and privacy not explicitly addressed	Security and privacy not explicitly addressed	Security and privacy not explicitly addressed
**Limitations**					
Scalability	Not explicitly discussed for large-to-massive scale deployments	Scalability of multi-agent system architecture needs evaluation	Scalability of semantic reasoning needs evaluation	Scalability challenges not explicitly addressed	Scalability of semantic reasoning for large-scale deployments
Real-time Search	Not explicitly addressed how efficiently it handles real-time data	Efficiency of handling real-time data with multi-agent communication needs evaluation	May introduce overhead due to semantic reasoning for real-time data	Real-time aspects not explicitly addressed	Real-time aspects not explicitly addressed
Standardization	Adherence to future WoT search standards is to be determined	Adherence to future WoT search standards is to be determined	Relies on establishing ontologies and semantic descriptions	Lacks emphasis on standardization	Lacks emphasis on standardization

**Table 2:** Complexity analysis of data structures.

Index Structure	Insert	Delete	Update	Search
Map *hash* and Linked List	O(1)	O(1)	O(1) *	O(n)
Map *hash* and B+ Tree	O(1)	O(1)	O(1) *	>O(Log n) **
Map *hash* and Red-Balck Tree	O(1)	O(1)	O(1) *	O(Log n)

Notes: (*) Update operation viewed as an insert + delete operation. (**) The search operation on publication lists can be optimally O(Log n), however, if the tree is not well balanced, the complexity can increase from O(h), tree height, to a maximum of n.

**Table 3:** IR Experimental XML collection of documents.

Entity Type	Entity Number	Document Siye (KB)	Size by Type (KB)
IZN	4	2.02	8.08
SSP	500	2.02	1010.00
SSS	16,500	1.27	20,955.00
VTH	16,500	1.13	18,645.00
VSN	16,500	1.13	18,645.00
Total	50,004	1.19	59,263.08

## Data Availability

Data are contained within the article.

## Abbreviations

The following abbreviations are used in this manuscript:

CoT	Cloud of Things
IoT	Internet of Things
IR	Information Retrieval
WoT	Web of Things
WoTSE	Web of Things Search Engine
XaaS	cloud-based services
